# Surgical treatment of retrorectal tumors: a plea for a laparoscopic approach

**DOI:** 10.1007/s00464-023-10448-5

**Published:** 2023-10-05

**Authors:** Clara Galán, M. Pilar Hernández, M. Carmen Martínez, Anna Sánchez, Jesús Bollo, Eduardo Mª Targarona

**Affiliations:** 1 Unit of Gastrointestinal Surgery, Service of Surgery, Hospital de Sant Pau, Autonomous University of Barcelona, Barcelona, Spain; 2 Institute for Biomedical Research, Hospital de Sant Pau (IIB Sant Pau), Barcelona, Spain

**Keywords:** Retro-rectal tumors, Laparoscopic transabdominal approach, Minimally invasive surgery, Perineal approach

## Abstract

**Introduction:**

Retrorectal tumors (RRTs) are rare and often surgically excised due to the risk of malignant degeneration and compressive or obstructive symptoms. The approach for excision has traditionally been based on tumor location and performed using either a transabdominal or perineal approach depending on the position of the tumor. The advent of minimally invasive surgery, however, has challenged this paradigm. Here, we determined the applicability and potential advantages of a laparoscopic transabdominal approach in a series of 23 patients with RRTs.

**Material and methods:**

We included 23 patients presenting with RRTs treated at the Surgical Gastrointestinal Unit at Hospital de Sant Pau that were registered prospectively since 1998. The preoperative evaluation consisted of colonoscopy, CT scan and/or MRI, mechanical bowel lavage, and antibiotic therapy. Signed consent was obtained from all patients for a laparoscopic transabdominal approach unless the tumor was easily accessible via a perineal approach. In case of recurrence, a transanal endoscopic microsurgery (TEM) approach was considered.

Surgical details, immediate morbidity, and short- and long-term outcomes were recorded.

**Results:**

Of the 23 RRT cases evaluated, 16 patients underwent a laparoscopic transabdominal approach and 6 underwent a perineal approach. No patients required conversion to open surgery. In the laparoscopic transabdominal group, the mean operating time was 158 min, the average postoperative hospital stay was 5 days, and postoperative morbidity was 18%. Three patients had recurrent RRTs, two of the three underwent surgical reintervention. The third patient was radiologically stable and close follow-up was decided.

**Conclusion:**

Our results show that laparoscopic transabdominal excision of RRT is a safe and effective technique, offering the potential advantages of less invasive access and reduced morbidity. This approach challenges the traditional paradigm of excision of these infrequent tumors based solely on tumor location and offers a viable alternative for the treatment of these infrequent tumors.

The retrorectal space is a potential site for infrequent types of histological lesions known as retrorectal tumors (RRT). Due to its embryological development, this virtual anatomic space joins several organs of varying histological origin [1, 2]. Although the origin varies, clinical features of RRT are similar. Growth inside the lower pelvis may be followed by local compressive or obstructive symptoms, which together with the potential risk of malignant transformation necessitate resection [1–3].

The classical rule of thumb for surgical excision is based on the location of the RRT. For tumors above an imaginary line crossing the pelvis at S2 or S3 sacral vertebrae a transabdominal approach is recommended, while for tumors located below this line, a perineal or a retrorectal approach is advised [2] (Fig. 1). Both approaches are highly invasive and the deep location of this lesion in the pelvis requires a wide dissection. Advances in the laparoscopic approach to rectal cancer and total mesorectal excision have facilitated knowledge of the anatomic features of the lower pelvis and its minimally invasive surgery (MIS). MIS access does not only dramatically reduce the invasiveness of the surgical approach but also breaks the old paradigm regarding the location of the tumor in relation to the S3 vertebrae [1–3]. Lower tumors are also accessible via the transabdominal route, thereby avoiding aggressive perineal or retrorectal incisions for access in many cases. However, due to the infrequent incidence of RRT, MIS experience regarding treatment for this disorder treatment is scarce. The aim of this study was to evaluate the potential advantages of the MIS approach in a prospective series of patients who underwent surgical resection of RRT.

**Fig. 1:**
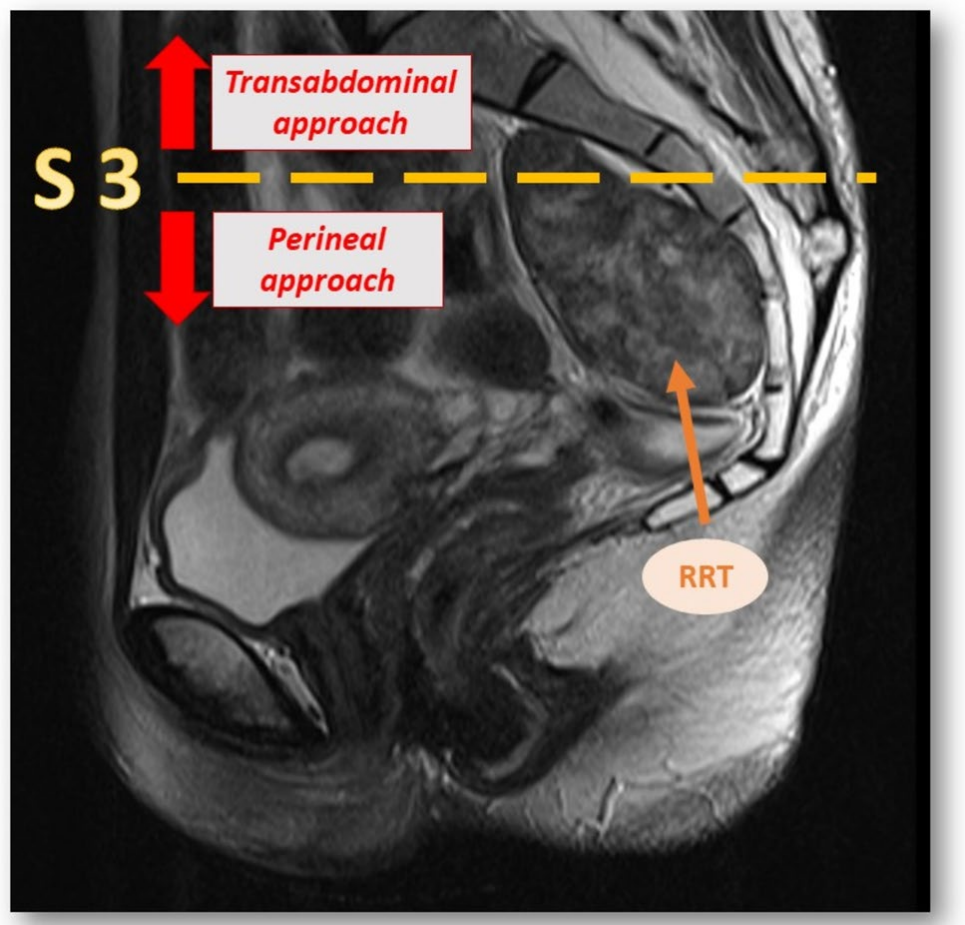
Classical thumb rule for surgical excision of the RRT based on the location above or below S3 sacral vertebra. Sagittal cross MRI of a RRT

## Materials and methods

Data regarding RRT at the Surgical Gastrointestinal Unit at Hospital de Sant Pau have been prospectively recorded since 1998 and were reviewed in April 2023. These data included all patients presenting with RRTs, defined as solitary lesions located extraperitoneally in the retrorectal space below the sacrum promontorium and not originating from digestive, urinary, or gynecological structures. We recorded patients’ age and sex, clinical symptoms, method of diagnosis, surgical details, and short- and long-term outcome. Cases in whom imaging techniques showed malignant diagnosis were excluded. A laparoscopic approach was offered in all cases unless the tumor was located in a position and of a size that facilitated easy access with a perineal approach (juxta or below the coccyx). In case of recurrence, a TEM approach was considered. We recorded the duration of the surgical procedure, length of hospital stay, and immediate morbidity (Clavien–Dindo classification). Following histopathological analysis of the surgical specimen, in cases of malignant findings, an oncological evaluation was made to determine whether further treatment was required. All patients had an annual follow-up and MRI or CT scan. This study did not require Institutional Review Board (IRB) approval.

### Surgical techniques

**Fig. 2:**
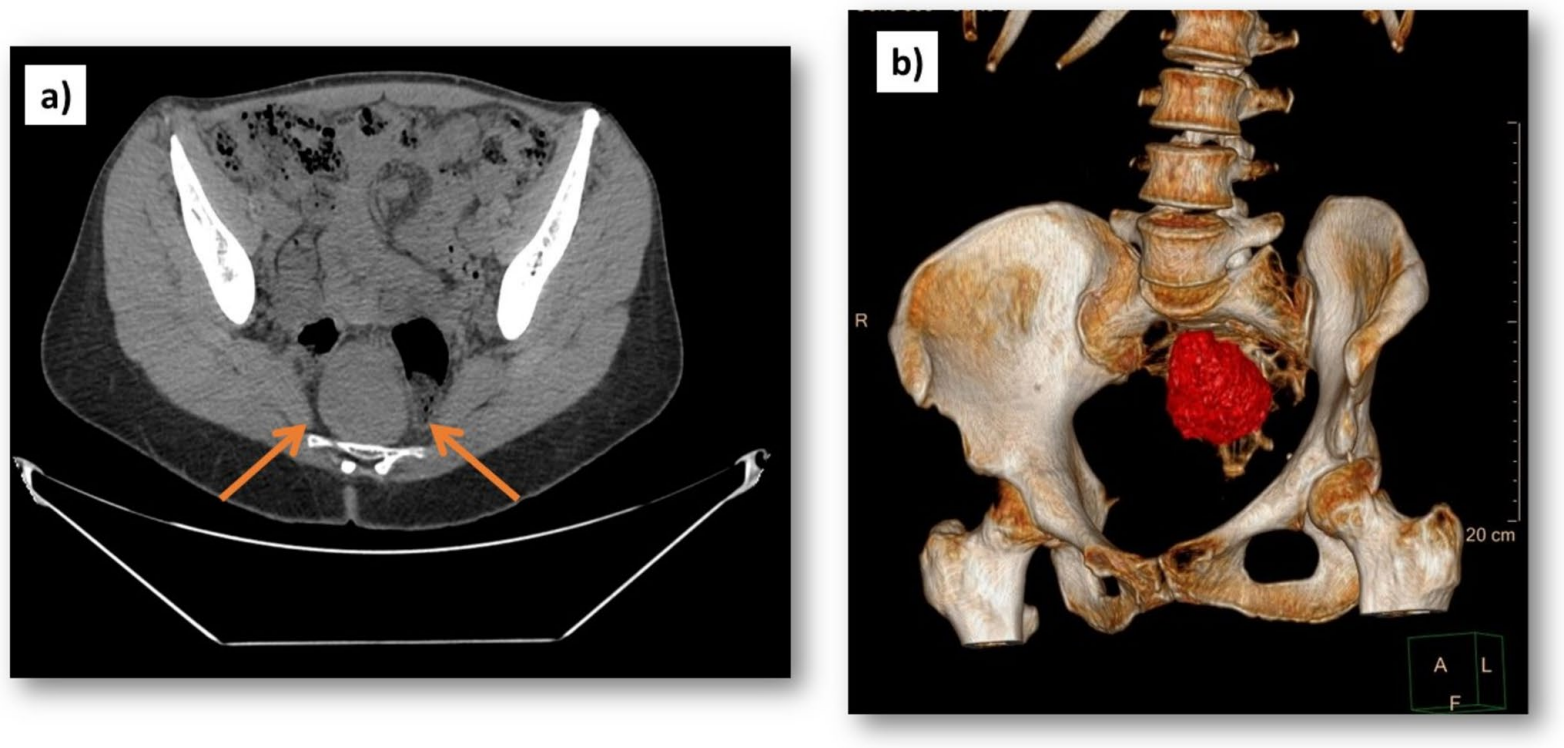
Preoperative abdomino-pelvic imaging test of a RRT: **a** cross sectional CT-scan; **b** 3D reconstruction of CT-scan

All patients were preoperatively evaluated by means of colonoscopy, CT scan, and/or MRI (Fig. 2). No patients had a preoperative biopsy of the lesion as part of the diagnosis. In the day before surgery, mechanical bowel preparation was performed. Surgery was performed under general anesthesia and endovenous antibiotic prophylaxis was provided.

### Transabdominal approach

**Fig. 3:**
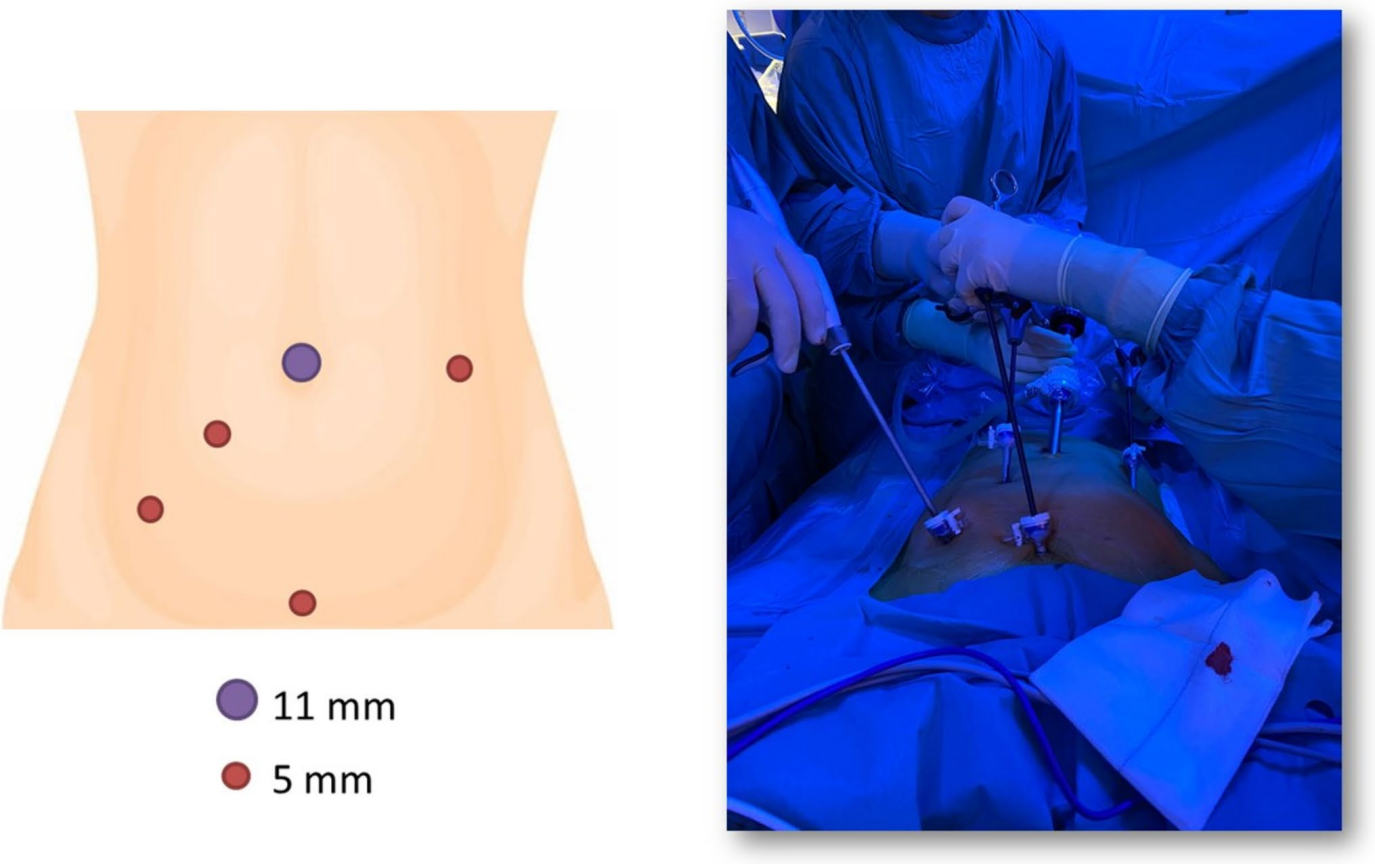
Image of a RRT excision with a transabdominal approach. Standard laparoscopy port positions are used

The patient was placed in a standard position for the lower pelvis laparoscopic approach, with legs on stirrups and with shoulders supported for the Trendelenburg position. Four trocars were used in all cases: a 10-mm trocar at the umbilicus, two 5-mm trocars on the right side of the lower abdomen, and one trocar on the left side (Fig. 3) [4]. Dissection began at the right pararectal gutter and the mesorectal space was located. The mesorectal space was opened and explored up to the upper pole of the RRT. Blunt and sharp dissections were performed until the tumor was completely dissected. When approaching the most distal part of the RRT, especially in cases located juxta the anorectal sphincter, a simultaneous digital rectum control was particularly useful to locate the specimen in order to rule out or reduce the potential risk of rectal wall injury. The tumor was extracted inside a bag and a Blake drain with low pressure was placed [4].

### Perineal approach

The patient was placed in a prone position, with the legs separated and the buttocks retracted. A vertical or transverse infra-coccygeal incision was performed, and the tumor was excised. Primary closure of the skin was performed.

### Transanal endoscopic microsurgery (TEM)

In the case of recurrent RRT, we used the standard TEM technique and the rectal mucosa was opened. Wide dissection of the recurrent cyst was performed, followed by closure of the rectal wall by means of a v-lock suture.

## Results

During the study period, 23 patients diagnosed as having RRT and eligible for non-extended surgery were evaluated for MIS excision.

There were 17 women and 6 men, with a median age of 58 years (range 27–86 years). Twenty-two of the 23 patients in the series underwent surgery. A closed follow-up was decided in the case of an older patient with a small lesion. One of the 22 patients who underwent surgery was reoperated for recurrent RRT via TEM. Sixteen patients (72%) underwent a transabdominal MIS. One of these patients presented a very low recurrent tumor and was treated with a TEM. Six patients (27%) with small (< 3 cm) and ultra-low juxta coccygeal lesions were approached through an open perineal route.

Table 1 summarizes the clinical features, lesion size, diagnostic methods, and preoperative clinical diagnosis. The immediate outcome after surgery is summarized in Table 2. In the MIS group, there were no conversions or intraoperative incidents other than accidental rupture of the cyst wall in four cases. Mean operating time was 158 min in patients treated by the laparoscopic approach and 45 min in patients treated by the perineal approach. The average postoperative hospital stay for all study patients was 4 days. Postoperative morbidity was low. There were three cases of local infection (deep infection in two and wound infection in one). These infections were treated with antibiotics and no aggressive measures were needed (Clavien–Dindo I) (Table 3).

**Table 1:** Description of cases: demography, clinical features and diagnosis

Case	Sex	Age (years)	Clinical feature	Diagnosis	Size (mm)
1	Male	64	Low abdominal pain	CT scan + MRI	57 × 52 × 51
2	Female	73	Low abdominal pain	MRI	40 × 15
3	Female	75	Casual diagnosis	CT scan + MRI	55 × 60 × 35
4	Female	72	Low abdominal pain	CT scan + MRI	26 × 22 × 15
5	Female	41	Low abdominal pain	CT scan + MRI	47 × 49
6	Female	75	Casual diagnosis	CT scan + MRI	79 × 36 × 25
7	Female	27	Low abdominal pain	CT scan + MRI	25 × 30 × 30
8	Male	55	Low abdominal pain	CT scan + MRI	23 × 20 × 30
9	Female	48	Casual diagnosis	CT scan + MRI	38 × 40 × 60
10	Female	33	Casual diagnosis	MRI	75 × 5 × 44
11	Female	59	Sciatic and lumbar pain	CT scan	60 × 50 × 30
12	Female	38	Casual diagnosis	CT scan	110 × 55 × 35
13	Male	84	Low abdominal pain	CT scan	70 × 60 × 40
14	Female	86	Low abdominal pain	CT scan	70 × 70 × 36
15	Male	63	Sciatic and lumbar pain	MRI	50 × 60 × 40
16	Male	40	Low abdominal pain	MRI	50 × 42 × 110
17	Female	32	Perianal abscess	CT scan	70 × 40 × 20
18	Female	43	Casual diagnosis	CT scan	35 × 25 × 40
19	Male	69	Casual diagnosis	MRI	45 × 68 × 30
20	Female	76	Low abdominal pain	MRI	30 × 16 × 20
21	Female	71	Casual diagnosis	CT scan	37 × 26 × 29
22	Female	58	Low abdominal pain	CT scan + MRI	25 × 20 × 20
23	Female	61	Casual diagnosis	CT scan + MRI	48 × 20 × 24

**Table 2:** Surgery approach, operating time and surgical complications

Case	Approach	Operating time (min)	Surgical complications
1	Laparoscopy	140	Accidental tumor rupture
2	Perineal	60	No
3	Laparoscopy	240	Accidental tumor rupture
4	Follow-up observation		
5	Laparoscopy	90	No
6	Laparoscopy + posterior transperineal (gluteal)	180	Accidental tumor rupture
7	Perineal	30	No
8	Perineal	30	No
9	Laparoscopy	180	No
10	Laparoscopy	90	No
11	Laparoscopy	75	No
12	Laparoscopy	145	No
13	Laparoscopy	180	No
14	Laparoscopy	190	No
15	Laparoscopy	200	No
16	Laparoscopy + posterior transperineal (sacro)	250	No
17	Laparoscopy + posterior transperineal	190	No
18	Laparoscopy	120	No
19	Laparoscopy	145	No
20	Perineal	60	No
21	Perineal	45	No
22	Perineal	50	Accidental tumor rupture
23	Laparoscopy	120	No

**Table 3:** Tumor characteristics, postoperative complications, hospitalization and recurrence

Case	Histology	Postoperative complications	Hospitalization (days)	Recurrence
1	Hamartomatous cyst (tail gut)	No	5	No
2	Hamartomatous cyst (tail gut)	No	4	No
3	Mucinous adenocarcinoma	No	7	Yes (reintervention: bilateral oophorectomy because of metastasis)
4	Follow-up observation			
5	Schwanoma	No	2	No
6	Mucinous adenocarcinoma	No	8	No
7	Desmoid cyst	No	1	No
8	Desmoid cyst	No	1	No
9	Heterotipia salivary glands	No	3	Yes (radiological estability)
10	Solitary fibrous tumor	No	4	No
11	Neurofibroma	Abscess	5	No
12	Teratoma	No	8	No
13	Teratoma	No	3	No
14	Hamartomatous cyst (tail gut)	No	4	No
15	Schwanoma	Abscess	14	No
16	Hamartomatous cyst (tail gut)	Wound infection	4	No
17	Hamartomatous cyst (tail gut)	No	3	Yes (reintervention: TEM)
18	Heterotipia salivary glands	No	4	No
19	Glomus tumor	No	3	No
20	Hamartomatous cyst (tail gut)	No	4	No
21	Hamartomatous cyst (tail gut)	No	3	No
22	Hamartomatous cyst (tail gut)	Abscess	3	No
23	Hamartomatous cyst (tail gut)	No	4	No

The definitive histological diagnosis is summarized in Table 3. Two cases were malignant (mucinous adenocarcinomas). One of these two cases received adjuvant therapy (chemotherapy and radiotherapy) without recurrence 5 years later and the other patient developed ovarian metastasis that required bilateral salpingo-oophorectomy. At long-term follow-up (60 ± 12 months), one patient who was previously treated with an MIS approach and transperineal resection developed symptomatic recurrence and TEM excision was performed. There was no reported follow-up mortality in our study.

Table 4 compares the main variables analyzed in our study between the two groups of patients treated with RRTs, the laparoscopic/combined approach versus the perineal approach.

**Table 4:** Variable comparison between patients with RRTs operated by laparoscopic or combined approach and patients operated by perineal approach

	Laparoscopy approach or combined approach	Perineal approach
Median age (years)	59.93 ± 17.41	60 ± 16.65
Median size (mm)	49.14 ± 9.73	25.74 ± 3.33
Median operating time (min)	158.43 ± 50.08	45.83 ± 12.38
Incidence of surgical complications	3/16 (18%)	1/6 (16%)
Pathological diagnosis (%)	Hamartomatous cyst (tail gut): 31.25% Mucinous adenocarcinoma: 12.5% Others: 56.25%	Hamartomatous cyst (tail gut): 66.66% Mucinous adenocarcinoma: 0 Others: 33.33%
Incidence of postoperative complications	3/16 (18%)	1/6 (16%)
Incidence of recurrence	3/16 (18%)	0
Median hospitalization stay (days)	5 ± 2.88	2.6 ± 1.24

## Discussion

In our series of patients with RRT, the laparoscopic approach was performed in 16 patients (72%, *n* = 16/22). The remaining 6 patients (27%) were treated by the perineal approach because they presented with small and distal tumors that were easily accessible by this route. Conversion to open surgery was not required in the minimally invasive surgery (MIS) group as there were no serious intraoperative complications. The patients clearly benefitted from the advantages of the MIS approach. The average postoperative length of the hospital stay was short (4 days) and there was a low incidence of postoperative morbidity (18%) (Table 4).

Two patients initially diagnosed with tail gut cysts were found to have mucinous adenocarcinoma after surgery in spite of no signs of malignancy were present at preoperative imaging.

The rarity of RRTs makes it difficult to have large series of patients and long-term follow-ups. Currently, there are a lack of quality information and experience regarding the management of this pathology, making it difficult to compare our results with other studies.

Table 5 shows the results of studies published in the last 5 years with more than 20 patients treated for retrorectal tumors [1–3, 5, 6]. If we compare our study results with these experiences, we can see that our series presents a higher number of patients operated by laparoscopy (72%, *n* = 16/22). Two of the most recent studies show the majority of patients underwent a perineal approach using transsacrococcygeal or transperineal technique because the tumors were located below the S3 sacral vertebra [1, 2].

**Table 5:** Literature review about surgical treatment of RRT (published in the last 5 years)

	Type of study	Period of study	*N* total	Sex	Median age (years)	Clinical features	Approach	Median size (cm)	Clavien Dindo classification	AP malignancy	Recurrence (*n*)	Median follow-up (months)
Ahmad Sakr et al. [5]	Retrospective	2007–2018	24	Females: 18 Males: 6	51.5	Symptomatic: 10 Asymptomatic: 14	Anterior laparoscopic: 10 Posterior: 11 Combined: 3	5.5 ± 2.7	CD II: 9 (37.5%) CD III: 1 (4.2%)	2: 1 neuroendocrine 1 adenocarcinoma	0	12
Orçun Yalav et al. [3]	Retrospective	2009–2019	20	Female: 12 Male: 8	48.3 ± 14.2	Symptomatic: 20	Anterior laparoscopic: 2 Posterior: 14 Combined: 4	5.4 ± 2.6	CD I: 25% CD II: 5% CD III: 5%	3: 2 chordoma 1 leiomyosarcoma	1	53.8 ± 40
Carpelan Holmström et al. [2]	Retrospective	2012–2017	52	Female: 40 Male: 12	43	Symptomatic: 22 Asymptomatic: 30	Anterior laparoscopic: 7 Posterior: 44 Combined: 1	*Max. tumor length:* *30*	CD I: 11	4: 1 malignant tailgut cyst 1 angiomyxoma 1 leiomiosarcoma 1 GIST	14	39
Zeyu Li et al. [1]	Retrospective	2009–2019	44	Female: 33 Male: 11	50	Symptomatic: 44	Anterior laparoscopic: 3 Posterior: 26 Combined: 2	5.6	CD I: 5 CD IIIa:1 CD Id: 1	18: 5 originated in presacral space 6 chordomas 2 teratomas 5 metastasic adenocarcinoma	9: benign tumors: 4 malignant tumors: 5	25
Mathilde Aubert et al. [6]	Retrospective	2000–2019	270	Female: 213 Male: 57	46 ± 15	Symptomatic: 151 Asymptomatic: 119	Anterior laparoscopic: 53 Abdominal open: 17 Abdominal robotic: 2 Posterior: 190 Combined: 8	5.7 ± 2.9	CD I–II: 56 CD III–IV: 16	22	20 benign tumors: 15 malignant tumors: 5	27 ± 39

Aubert et al. published the largest multicenter study to date on the management of RRTs [6]. A total of 270 patients operated for RRT in 18 academic French centers were retrospectively included from 2000 to 2019. Of these, 27% (*n* = 72) underwent the laparoscopic anterior approach, 70% (*n* = 190) the posterior approach, and 3% (*n* = 8) a combined approach. Laparoscopy was frequently performed for RRTs that were symptomatic, large, or located above S3 vertebra. The authors concluded that these features may explain the poorer intraoperative results and the higher conversion rate and longer operative time. They did not report any significant difference between the two surgical approaches in terms of morbidity, reintervention, readmission, or recurrence. The results of the study by Aubert et al. [6] are comparable to our study results regarding the period in which it was carried out, and it is indicative of how the laparoscopic approach is not the most commonly used approach today for the treatment of these tumors.

Additional surgical approaches are currently available and feasible for this type of tumors, such as minimally invasive transanal surgery/transanal endoscopic microsurgery (TAMIS/TEM) and robot. TAMIS or TEM are alternative approaches for surgical treatment of RRTs. Both methods provide safe tumor excision and a short operative time, low morbidity, and better postoperative recovery. The TEM approach was first described by Zoller et al. in 2007 [7], and studies with short series of patients suggest that despite its technical demands, the results are attractive, especially for the deep pelvis and cystic lesions with benign appearance, due to low intraoperative and postoperative complications [8–11]. In our study, a case initially operated with laparoscopic surgery had a distal recurrence and a rescue TEM was performed. A systematic review by Mullaney et al. [12] included 82 patients, with 73 patients undergoing laparoscopic or combined laparoscopic and perineal approaches and 9 patients received robot-assisted surgery. The review found that robotically operated patients had longer operative times but postoperative outcomes similar to those for laparoscopic surgery patients [12]. Other smaller studies have reported shorter operative times, less blood loss, and shorter hospital stays in patients who underwent robotic surgery [13]. Despite not having any robot-operated patients in our study, our opinion is, the robot can be considered a good surgical approach for tumors located in the deep pelvis because it allows better vision and aids manipulation of the deep pelvis structures.

An important issue in the management of RRTs is the risk of malignant changes. This risk is variable and depends on of the type of the tumor. Several studies have reported varying incidences of malignancy, with Sakr et al. [5] and Mathis et al. [14] reporting incidences up to 8% and 13%, respectively. A recent systematic review by Nicoll et al. [11] in 2019 found an overall rate of neoplastic transformation of 26% in tail gut cysts. Another systematic review in 2020, by Feng Liang et al. [15], reported that up to 30% of tail gut cysts in the literature were malignant in the literature.

Long-term follow-up of retrorectal tumors (RRTs) is mandatory due to the possibility of recurrence. Recurrence rates are generally lower for benign tumors (1–2%) than for malignant tumors (30–50%) [2]. In our study, three patients presented recurrence, two with a benign RRT and one with a malignant RRT. Two of these patients underwent re-operation. The third patient was radiologically stable and close follow-up was decided.

The main limitation of our study is that due to the rarity of the RRTs, the number of cases is limited. Further research with a multicenter prospective design could help determine the long-term effectiveness of the laparoscopic approach to RRT.

The traditional surgical approach for rectal resection and tumor treatment for RRTs involves wide, invasive incisions, either through the transabdominal or transperineal/retrorectal approach, following the S3 vertebra rule [16, 17]. However, advances in minimally invasive surgery (MIS) techniques have allowed refinement of the surgical approach for lower rectum and deep pelvis conditions, such as rectal cancer and prolapse. In our practice, tumors located below S3 are not considered for a direct perineal approach. The posterior approach is our preferred option only for very small and easily accessible perineal lesions. We believe that laparoscopy should be considered a feasible option in suspected cases of malignancy without neighboring structure invasion, provided care is taken for complete tumor resection to avoid rupture of the wall of the tumor. This approach can avoid the more aggressive open alternative approaches.

## Conclusion

RRT are rare tumors that grow in the retrorectal space. Surgical resection is indicated due to the potential risk of malignancy. Surgery has classically been performed by the posterior approach. However, with the new laparoscopic techniques, MIS provides a safe and feasible alternative for resection of this type of tumors.
